# A randomized clinical control study on the efficacy of three-dimensional upper limb robotic exoskeleton training in chronic stroke

**DOI:** 10.1186/s12984-022-00991-y

**Published:** 2022-02-04

**Authors:** Antonio Frisoli, Michele Barsotti, Edoardo Sotgiu, Giuseppe Lamola, Caterina Procopio, Carmelo Chisari

**Affiliations:** 1 Institute of Mechanical Intelligence, Scuola Superiore Sant’Anna of Pisa, PERCRO Lab, Via Alamanni, 13b, San Giuliano Terme, Ghezzano, 56010 Pisa, Italy; 2 INL-International Iberian Nanotechnology Laboratory, Braga, Portugal; 3 University Hospital of Pisa, Pisa, Italy

**Keywords:** Stroke, Exoskeleton, Robotic rehabilitation training, Robotic biomarkers

## Abstract

**Background:**

Although robotics assisted rehabilitation has proven to be effective in stroke rehabilitation, a limited functional improvements in Activities of Daily Life has been also observed after the administration of robotic training. To this aim in this study we compare the efficacy in terms of both clinical and functional outcomes of a robotic training performed with a multi-joint functional exoskeleton in goal-oriented exercises compared to a conventional physical therapy program, equally matched in terms of intensity and time. As a secondary goal of the study, it was assessed the capability of kinesiologic measurements—extracted by the exoskeleton robotic system—of predicting the rehabilitation outcomes using a set of robotic biomarkers collected at the baseline.

**Methods:**

A parallel-group randomized clinical trial was conducted within a group of 26 chronic post-stroke patients. Patients were randomly assigned to two groups receiving robotic or manual therapy. The primary outcome was the change in score on the upper extremity section of the Fugl-Meyer Assessment (FMA) scale. As secondary outcome a specifically designed bimanual functional scale, Bimanual Activity Test (BAT), was used for upper limb functional evaluation. Two robotic performance indices were extracted with the purpose of monitoring the recovery process and investigating the interrelationship between pre-treatment robotic biomarkers and post-treatment clinical improvement in the robotic group.

**Results:**

A significant clinical and functional improvements in both groups (p < 0.01) was reported. More in detail a significantly higher improvement of the robotic group was observed in the proximal portion of the FMA (p < 0.05) and in the reduction of time needed for accomplishing the tasks of the BAT (p < 0.01). The multilinear-regression analysis pointed out a significant correlation between robotic biomarkers at the baseline and change in FMA score (R^2^ = 0.91, p < 0.05), suggesting their potential ability of predicting clinical outcomes.

**Conclusion:**

Exoskeleton-based robotic upper limb treatment might lead to better functional outcomes, if compared to manual physical therapy. The extracted robotic performance could represent predictive indices of the recovery of the upper limb. These results are promising for their potential exploitation in implementing personalized robotic therapy.

*Clinical Trial Registration* clinicaltrials.gov, NCT03319992 Unique Protocol ID: RH-UL-LEXOS-10. Registered 20.10.2017, https://clinicaltrials.gov/ct2/show/NCT03319992

## Background

Upper limb motor impairment is one of the most frequent causes of long term disability following stroke and it is particularly problematic given its negative impact on Activities of Daily Living (ADL) [1]. Physical therapy and exercise promote the motor recovery after stroke with consequent regain of function and changes in cortical reorganization according to residual neuroplasticity [2]. It has been demonstrated that the amount and intensity of practice and the degree of participation, as well as the task-oriented training, play a crucial role in positively affecting the neuroplastic changes [3]. Apart the intrinsic ability of providing a high number of specific practice movements, robot-mediated therapy can be successfully coupled with virtual reality (VR) technology allowing patients to train in a more ecological and enriched environment which could give an opportunity to practice functional movements and everyday activities that are not or cannot be practiced within the hospital environment [4].

However, although scientific literature provides supporting evidence of the efficacy of upper limb robotic treatments after stroke compared to manual therapy [5, 6], it is still arguable the achievement of an effective improvement in terms of regained upper limb function and consequent transfer of abilities to ADL. One recent, large pragmatic randomized controlled trial performed with the MIT-Manus robotic gym system [7] concluded that robot-assisted training did not lead to improvement in upper limb function in ADLs compared with usual care, measured by ARAT test. To overcome this potential limit of some robotic rehabilitation programs, it has been so far hypothesized in literature that robotic training with exoskeletons, based on three-dimensional spatial, task-oriented and more naturalistic movements [8], is likely to provide higher benefits in terms of recovery in ADLs and improvement of upper limb function.

In the scientific literature there are still however not only a limited number of randomized controlled trials (RCT) concerning robotic therapy with three-dimensional spatial robotic exoskeletons to support this hypothesis, but also contrasting evidences. The asymmetry of studies conducted with End Effector (EE) devices vs Exoskeletons (Exo) is for example evident from data published in one recent meta-review [5], where only 3 exoskeletons RCT are reported, of which one based on passive exoskeleton device only,vs. 11 trials employing EE devices.

One of the first clinical studies addressing this issue was the randomized trial conducted with Pneu-WREX in a group 26 patients [9], wherein three dimensional movement against gravity in the context of simulated functional tasks that required use of the hand conducted with assist-as-needed controller robotic training was found to be more effective than conventional table-top training. According to authors’ the observed results benefits may also have arisen in part due to the fact that the robot allowed 3D movements that incorporate hand grip and release, rather than just planar or single-joint movements.

In a large controlled study (77 patients) [10], the robotic treatment conducted with the ARMin exoskeleton was compared with the manual physical and occupational therapy, showing that robotic training enhanced arm motor function more effectively than manual therapy, as measured by the upper extremity portion of the Fugl-Meyer scale (FMA-UE).

Also we observed in our previous study [11] in chronic stroke through instrumental study of the reaching performance that exoskeleton training produced positive effects in movement execution, in terms of decreased execution time, improved movement smoothness and increased active joint ranges of motion.

On the other side, another recent randomized controlled trial compared End Effector (EE) and Exoskeleton (Exo) robot therapy in patients with stroke [12] after 4 weeks of intervention, suggesting that the EE robot intervention is better than the Exo robot intervention among chronic stroke patients with moderate-to-severe impairment of upper extremity function.

Also within the cross-over study conducted with BONES exoskeleton [13], patients were assigned with different random order to both single joint and multiple joint robotic training. The results of the study showed how multi-joint functional robotic training was not superior to single joint robotic training for Box and Block Test score (primary outcome) and for other secondary outcome measurements (FMA, Wolf Motor Function Test WMFT, Motor Activity Log MAL scales).

So to what extent the 3D nature of therapy robotic assistance provided with exoskeletons can be a determinant factor for motor recovery?

To provide further clinical and experimental evidences to answer this question, we have compared within a randomized controlled clinical trial the effects of a robotic exoskeleton training in three-dimensional task-oriented exercises versus an equally intensive program of manual therapy intervention (1) to assess if the observed motor improvements are reflected into higher functional outcomes—and so improved transfer of abilities into ADL—than conventional manual therapy and (2) to understand how the eventual observed changes can be interpreted in terms of kinematic measurements automatically extracted by the exoskeleton.

As a second aspect, several clinical studies, including animal ones [14], support with growing consensus that individualized approach to stroke rehabilitation, for instance based on stratification of patients into groups with different probabilities of upper limb recovery, could enhance the recovery of lost motor function. In this context, the use of biomarkers plays an important role [15, 16]. Beside neurophysiological and neuroimaging biomarkers, robotic biomarkers may be a valuable clinical instrument for determining the effect of a rehabilitation therapy [17]. These robotic biomarkers have the great advantage to be entirely objective in capturing the quality of movement which can be immediately provided as an index of the recovery progress [18]. The extraction and the analysis of robotic biomarkers can be used for both monitoring the ongoing recovery process during treatment and for investigating the relationship with primary clinical outcome. It is reasonable to think that as next step robotic biomarkers can be used to optimize the design of rehabilitation therapies tailored to the need of individual patients.

In [19] first, it was demonstrated the high potential of prediction of the outcome of a therapeutic treatment in stroke, performing an objective and analytical assessment of motor recovery through the acquisition of kinesiological and kinetic parameters and finding a prediction, supported by a statistically significant correlation with clinical scales, while in [20] it was confirmed the capability of predicting Fugl-Meyer assessment scale through robotic and clinical biomarkers.

Predictive clinical models of post-stroke motor recovery allow specific early interventions which is the phase in which the largest treatment effect can be obtained. Patient-specific prognostic models for monitoring post-stroke recovery have been developed and validated to assess their clinical effectiveness [21]. The most reliable predictors of the functional outcome are age and motor function assessed on clinical scales immediately after the acute event [22].

The use of metrics based on biomechanical parameters to estimate movement capabilities can raise the knowledge about motor recovery mechanisms. However, because of insufficient validation, the clinical integration of those methods is still limited.

So based on the characteristics of spatial movement involved in exoskeleton rehabilitation, a secondary goal of the study is to investigate whether in the robotic group the measured robotic performance biomarkers, based on patient’s performance automatically extracted at the enrollment of treatment, could predict the clinical and functional outcome of the robotic rehabilitation treatment.

## Methods

### Study aim and design

The study was based on a Parallel-Group Control Randomized Trial. Patients were assigned with simple randomization [23] to two different intervention groups, namely, the manual physical therapy (the control group, CG) and the robotic-aided therapy (the robotic group, RG).

The robotic therapy was administered by means of the L-EXOS robotic exoskeleton coupled with specifically designed virtual reality rehabilitation exercises. Moreover, within the RG, the prediction ability of the robotic metrics measured at the enrollment of the patients to estimate the change in the FMA assessment after therapy was investigated.

Primary outcome measure of the study was the Fugl-Meyer Assessment (FMA) scale restricted to upper extremity.

To evaluate the impact of training in terms of transfer to ADLs and upper limb functional outcomes, as secondary outcome a functional assessment called Bimanual Activity Test (BAT) was used, consisting in the evaluation—in terms of execution time and quality of movement—of a variety of gross and fine bimanual manipulation tasks, which are the basis for many of the Occupational Tasks and ADL (details are reported in “Functional assessment” section). Moreover only in the Robotic Group, robotic performance biomarkers were extracted and computed at each session and analyzed post-treatment to assess whether they can predict the clinical and functional outcome of treatment.

### Participants

In order to minimize the confounding effects of spontaneous recovery only chronic patients were enrolled in the study. 26 unilateral hemiparetic chronic stroke patients were recruited from the pool of outpatients of the Neurorehabilitation Unit of the University Hospital of Pisa, but only 22 completed the treatment as detailed in “STUDY PARTICIPATION” section (aged 65.8 ± 11.3, 7 females and 15 males). The sample size was chosen based on the Cohen’s d index of d = 0.65 from previous studies of upper limb rehabilitation in stroke [24].

All recruited patients were right handed and they had a left ischemic or hemorrhagic cerebrovascular accident (21 and 5 respectively) at least 7 months before the beginning of the experiment. All patients provided written informed consent for participating in the study that was approved by the Ethical Committee of the AOUP (NCT: 03319992) and in compliance with the principles of the Declaration of Helsinki.

Patients were considered eligible for the study if they met the following inclusion criteria: (1) age ranged between 30 and 80 years; (2) diagnosis of a first-ever left hemisphere ischemic or hemorrhagic stroke at least 6 months prior to entry into the study; (3) minimum ability for shoulder humeral elevation; (4) upper-extremity motor function FMA score ≥ 15 (out of 66); (5) absence of neurological or muscular disorders that interfere with neuromuscular function; (6) absence of severe cognitive deficits that would limit patients’ ability to complete the study; (7) minimum score of 2 in the Modified Ashworth Scale; (8) not participating in any experimental rehabilitation or drug studies at the same time and (9) no previous experience with robotic treatments.

### Procedure

**Fig. 1:**
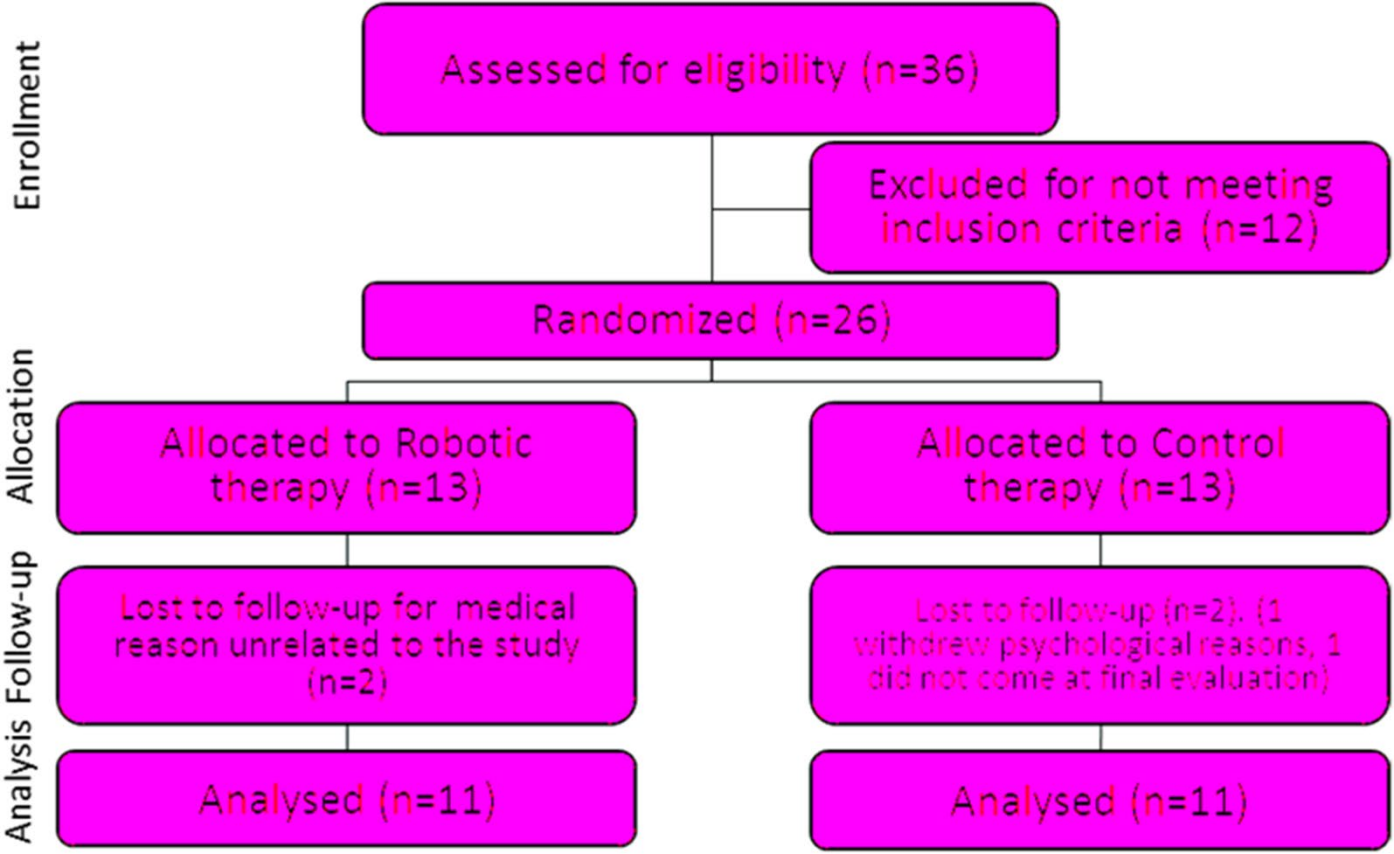
Consort Diagram of the Clinical Trail

The robotic and manual physical therapy treatments were equally matched in terms of intensity and duration. Patients belonging to both groups (CG and RG) performed 3 weekly rehabilitation sessions, of at least 45 min each, over a period of 6 weeks, with clinical evaluations at the enrollment and discharge (Fig. 1).

#### Manual intervention

**Fig. 2:**
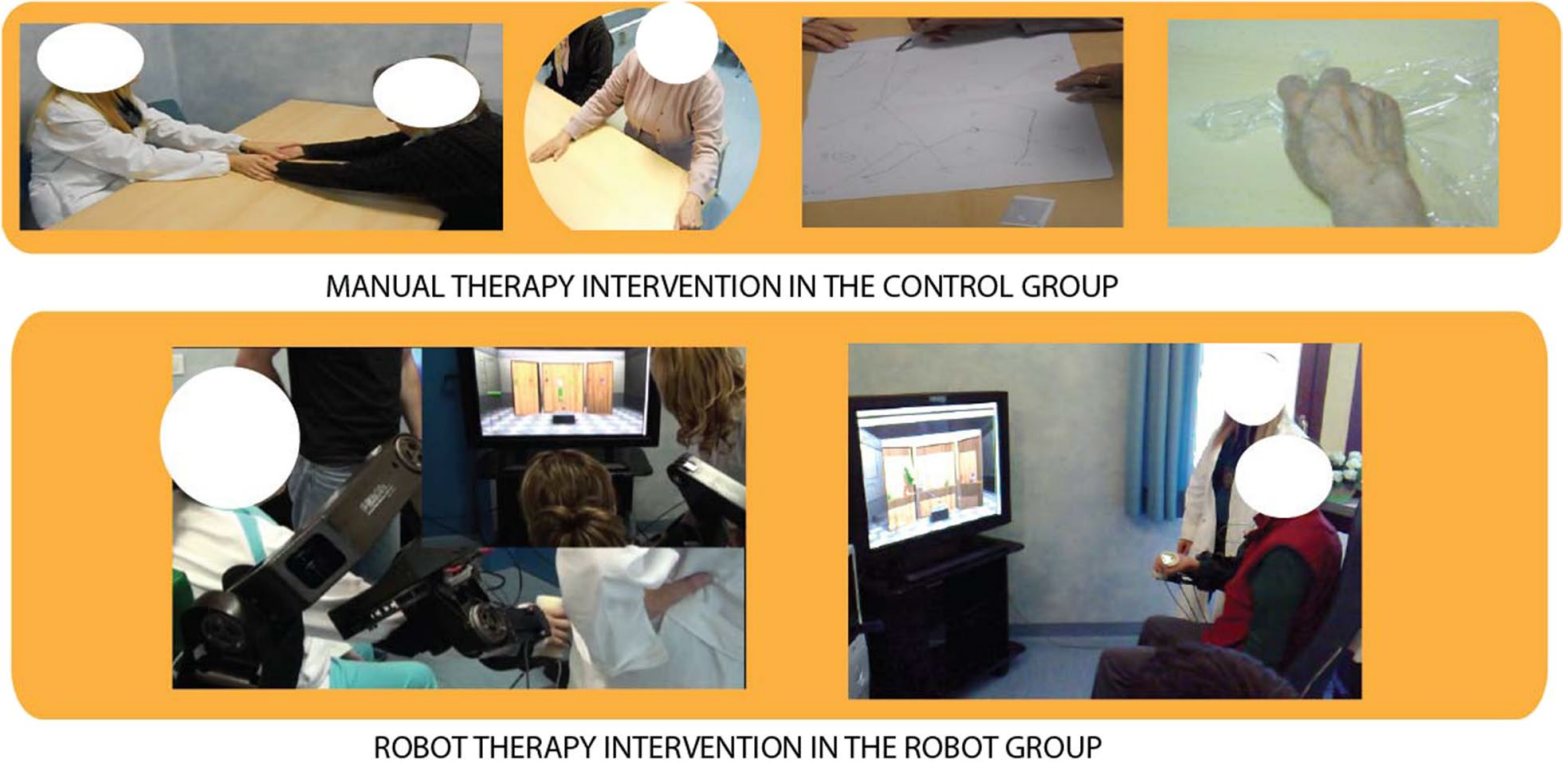
Manual therapy vs robot therapy intervention

The manual rehabilitation sessions consisted in physical therapy exercises mainly focused on reaching and grasping tasks using the affected limb and tailored to the need of each patient (see Fig. 2 top).

In order to maintain a comparative approach to the rehabilitation program, the following points were kept also in the physical therapy program.

*Passive movement* Passive movement and stretching of patient’s upper extremity was performed at the beginning of each session by the therapist.

*Goal directed movement and voluntary action* Exercises were proposed by the therapist according to the different level of motor impairment requiring upper extremity movement, visuo-motor coordination and sensory stimulation consisting in reaching and manipulation of objects with different consistency (plastic film, paper, common objects) to stimulate somatosensory afferents, contour following with pen and paper and 3D spatial movements to stimulate upper limb proprioception according to the Perfetti’s neurocognitive approach [25], and pick and place with peg wooden puzzles to replicate the tasks proposed in the robotic group.

Comparing the experimental robotic treatments vs. the control group, a higher involvement of hand function was present in the physical therapy group, while higher intensity and amplitude of arm movement (shoulder and elbow joints) was present in the robotic group.

#### Robotic intervention

Patients performed two training and one evaluation exercises at each robotic assisted rehabilitation session.

Patients sat comfortably on a chair in front of a 46 inches LCD screen placed at the distance of 1 m, wearing stereoscopic glasses and the L-EXOS exoskeleton [26] on their right (impaired) upper limb. For patients sitting on their own wheel chair, the right armchair was removed in order not to interfere with the L-EXOS. The height of the L-EXOS was adjusted to comfortably fit and properly support patient’s upper limb.

The L-EXOS is a tendon-driven robotic exoskeleton characterized by a serial kinematics consisting of five rotational joints, of which the first four actuated: kinematically the first three rotational axes are incident and mutually orthogonal (two by two) in order to emulate the kinematics of a spherical joint with the same center of rotation of the human shoulder, while the fourth axis is assumed coincident with the elbow joint and the fifth axis with the forearm (only sensorized), in order to allow the prono-supination of the wrist. The L-EXOS features a remote placement of electrical motors in order to drastically reduce the perceived inertia during free movement, by using tendon transmission that can easily transmit torques to joints placed far apart from motors with zero backlash, low friction and low weight. Thanks to these technical adopted solutions the L-EXOS can provide a reliable and smooth measurement of both joint and end-effector position.

The two training exercises were presented in a virtual reality simulated environment, specifically designed for the recovery of reaching and manipulation functions in stroke, and they were executed under the adaptable assistance of the robot. The exercises were designed with the aim of reproducing functional tasks of reaching, requiring visuo-motor coordination and involvement of spatial movement of the arm.

**Fig. 3:**
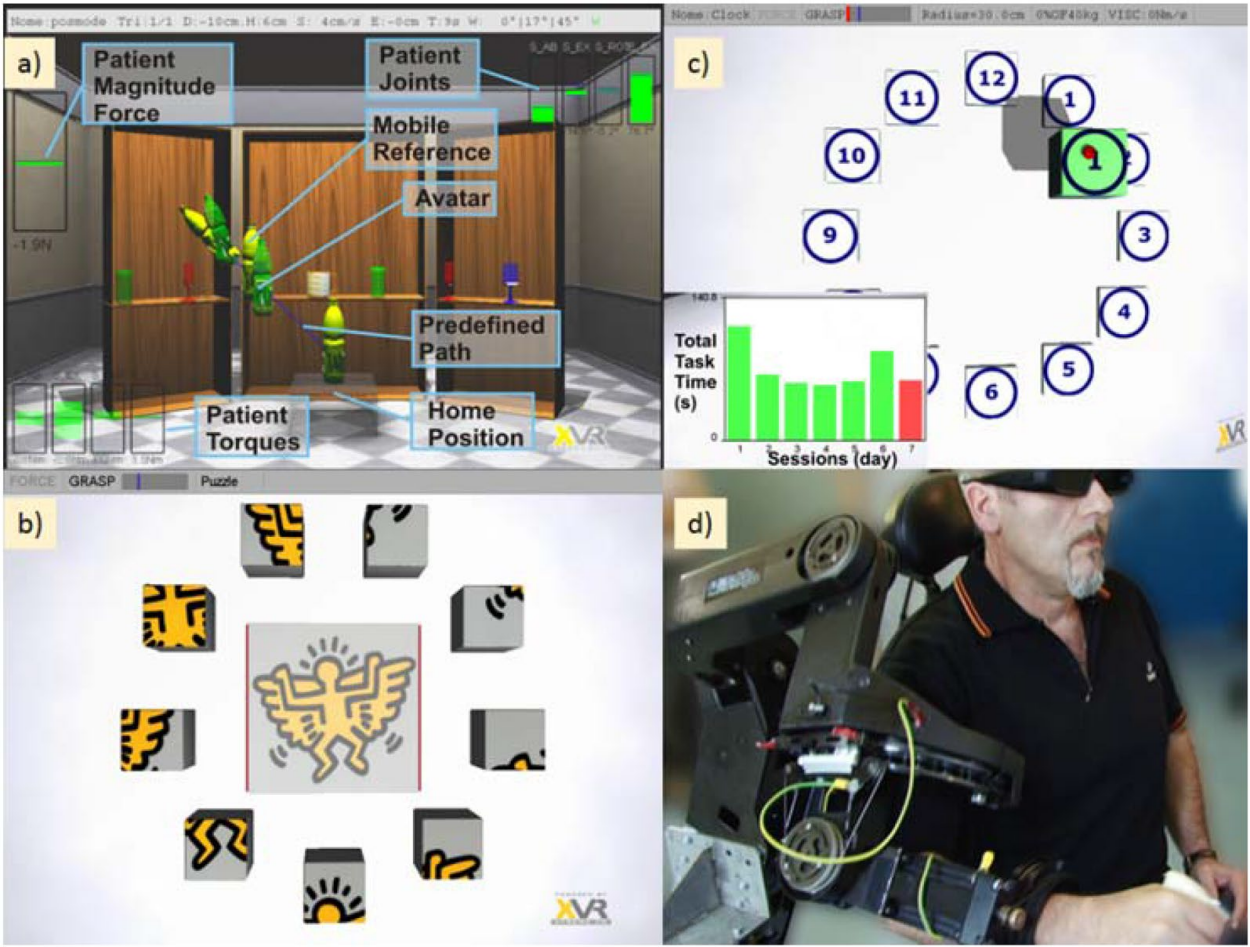
Robotic setup overview. a and **b** depict the Robotic Rehabilitation Games whereas **c** shows the robotic evaluation exercise. Inset **d** represents a patient during the robotic treatment

The first exercise (Fig. 3a) consisted of a reaching task with robotic assistance in which e patients were asked to virtually pour water into a set of glasses and cups. In particular, the virtual environment showed a number of glasses and cups placed on the shelf of a wide cupboard, at different height and positions (ipsilateral, contralateral, central positions with respect to the impaired arm), while patient’s hand was represented as a bottle. At the beginning of each trial, a target glass to be reached was highlighted as well as the line trajectory for reaching it. The patient was then asked to reach the target and pour the water by prono-supinating his/her wrist and, finally, to come back to the initial position. Task difficulty was varied according to patient status and condition, changing the distance and placement of target to be reached.

In the second exercise, the patient had to compose a virtual puzzle (see Fig. 3b). The patient was asked to reach and grasp each block placed symmetrically at twelve equally spaced positions around a circle on a vertical wall and place the block at the right place in the figure displayed at the center, to match the corresponding image. This task required a cognitive load to identify the correct target location of each block, according to the recognition of image displayed on the block face.

Two kinds of assistance were provided by the robot according to the task: an adjustable gravity counterbalancing of weight of the patient’s arm to relieve own weight and a guided assistance, according to an impedance-based model that actively assist the patient’s movement towards a selected target (for a detailed explanation of the exercises see [27]). In both exercises the difficulty of sessions was tailored to the patient’s ability and performance by the therapist using a user-friendly graphic interface.

The final part of each robotic rehabilitation session consisted of an evaluation exercise (see Fig. 3c) specifically designed for collecting performance indexes about the ability of the patient.

### Assessment procedures

#### Clinical assessments

Clinical evaluations of participants with stroke were administered by clinical specialists and physical therapists, with at least ten-year experience, involved in the study.

The primary outcome measure of the study was the motor function domain of the upper extremity portion of the Fugl-Meyer Assessment (FMA, 66 points score). Other clinical assessments included the modified Ashworth (MA) scale and a functional evaluation of upper limb by means of the Bimanual Activity Test (BAT).

The FMA was further analyzed in terms of sub-items. In particular, the motor FMA score was divided into proximal (shoulder and elbow movement, 36 points) and distal (hand and wrist movement, 24 points) sub-items. The BAT data were divided into pinch-tasks and power-tasks collecting those items requiring fine and gross manipulation motor skills respectively.

#### Functional assessment

The BAT scale was specifically designed to quantify the contribution of patient’s affected upper limb to execute common ADLs. The test assesses both execution time and quality of execution based on the assumption that complex upper extremity movements used in ADL are composed of several movement patterns (e.g., supination/pronation, grasp/release, pinch grip, etc.). The execution time was measured in milliseconds and the quality of execution scores on a 4 points scale. The scale consisted of 25 items matched to the corresponding tasks reported in Table 1. The items were grouped into pinch and power tasks according to the fine and gross manipulation motor skill respectively required for the execution. The selected list of test items represented upper limb movements necessary to perform many of the ADL.

**Table 1:** Items of bimanual activity test (BAT)

#	Task	Type	#	Task	Type
1	Loosen and tighten the cap of a bottle	Power	14	Open and close a zip	Pinch
2	Open and close a padlock	Power	15	Fast and unfasten a belt buckle	Pinch
3	Cut a piece of modeling paste using fork and knife	Pinch	16	Squeeze the toothpaste on a toothbrush	Power
4	Loosen and tighten the cap of a 10 cm jar	Power	17	Spread a tablecloth over a table	Power
5	Tear a piece of paper in four parts	Pinch	18	Roll a poster and close it with an elastic	Power
6	Draw a line using a pencil and a ruler	Pinch	19	Unscrew a bolt	Power
7	Cut a piece of paper in two parts using scissors	Pinch	20	Open a safety closure cap	Power
8	Open a closed paper-bag	Pinch	21	Open a glasses case	Power
9	Fold a piece of paper and place it in a paper-bag	Pinch	22	Open a pack of handkerchief and take one	Pinch
10	Staple 2 pages	Pinch	23	Move a 1 kg shoe box	Power
11	Tie a bow on a gift box	Pinch	24	Move a shoe box over another shoe box	Power
12	Tie a shoe	Pinch	25	Move a ball from the ground to a table	Power
13	Shuffle playing cards	Pinch			

Different background colors highlight the sub-division in Pinch and Power tasks (fine mobility and gross movements)

#### Robotic measures

Only RG patients were further assessed at the end of each session through an evaluation exercise performed without robot assistance and the analysis of their kinesiological performance during the task execution, based on the recorded kinematic parameters, e.g. hand position, velocity and upper extremity joint velocities, associated to the performed movement. The patients were instructed to reach different targets positioned in front of them and placed around a vertical circumference at 12 equally spaced locations. This configuration allowed to assess the reaching performance towards target locations in different portions of the peri-personal space (contralateral, mid, and ipsilateral with respect to the side of motor impairment) in terms of smoothness and execution time. The proposed task required the inter-articular coordination of both shoulder and elbow joints and support against gravity movement to reach elevated targets, representing a potentially useful exercise for evaluating the recovery of inter-joint coordination and abnormal movement synergies, i.e., elbow flexion associated to shoulder abduction, in reaching.

Two robotic measures were extracted for each outgoing (from the center to the target) movement: the execution time and the smoothness. The execution time was measured as the elapsed time for accomplishing each movement, measured from the time of grasping of the virtual object at the start position to the release time at the target position. The smoothness index was computed in the same interval period by counting the number of peaks in the velocity profile of movement, namely the Number of Movements Units (NMU) [28]. More in detail, a peak was counted if the difference from a minimum to the next maximum of the norm of the tangential speed was above 15% of the global maximum speed. In this exercise, the grasping and releasing of the virtual object took place automatically when the virtual avatar was over the object and the target respectively, without requiring any force at the level of the handle. In the subsequent analysis, the vertical plane, where targets were placed, was divided into two identical sub-plane containing 5 targets for the ipsi-lateral movements (targets from “1” to “5” in Fig. 3c) and 5 targets for the contra-lateral movements (targets from “7” to “11” in Fig. 3c).

### Statistical analysis

Differences in type of lesion, months post-stroke, age and gender between groups were evaluated with Mann–Whitney U (continuous and ordinal data) and Chi-square tests (categorical data). The outcome measures were analyzed using a 2-way mixed ANOVA with evaluation time (Pre and Post therapy) as the repeating factor and group (Robotic Vs. Control) as the between subjects’ factor. When significant interaction was detected, analysis of main effects was performed. Normality of the distribution of the outcome measures was assessed by means of the Lilliefors test and homogeneity of variance between groups was assessed through the Levene’s test.

The ability of predicting the change pre- and post-therapy in the FMA score using the robotic performance measured at baseline (pre-therapy) was investigated through a multilinear regression analysis, with the execution times $t_{ipsi}$ and $t_{contra}$ and the smoothness indicators $s_{contra}$ and $s_{ipsi}$ of both ipsilateral and contra-lateral movements as the predictor variables and the change in the FMA as the response variable.

$$\Delta \widehat{FM}=c+\sum_{i=ipsi,contra}({a}_{i}{t}_{i}+{b}_{i}{s}_{i})$$

The F-test on the regression model was used for assessing the significance of the linear regression relationship between the response variable and the predictor variables.

## Results

### Study participation

Table 2 reports the characteristics of the patients who completed the whole rehabilitation training divided by groups. As shown in the Consort flow diagram in Fig. 1, four patients (15%) withdrew and did not complete the final evaluations, so they were not included in our analysis. Two of them withdrew because of medical reasons unrelated to the study, one for psychological reasons and one did not come at the final evaluation.

**Table 2:** Patients information by group

	Robotic group	Control group
Gender	11; 4 females/7 males	11; 3 females/8 males
Age	62 ± 12 years	70 ± 11
Months post-stroke	30 ± 20 (min 7)	37 ± 24 (min 8)
Type of stroke	2 Hemorragic; 9 Ischaemic;	3 Hemorragic; 8 Ischaemic

### Clinical outcomes

Table 3 reports all observed changes in clinical outcome measures after treatment compared to the baseline values measured before treatment.

**Table 3:** Changes in clinical outcome measures

Outcome measure	Group	Baseline	Changes after therapy	p of change within groups	p between groups at baseline	p of changes between groups	p ANOVA main effect
FM	CTR	26.7 ± 16.3	8.9 ± 17.6	< 0.01**	0.86	0.46	< 0.01**
	ROB	25.6 ± 12.3	11.1 ± 13.9	< 0.01**			
FM (proximal)	CTR	19.3 ± 11.0	2.6 ± 10.9	< 0.05*	0.74	< 0.05*	< 0.01**
	ROB	18.0 ± 6.6	6.9 ± 7.8	< 0.01**			
FM (distal)	CTR	7.5 ± 6.6	6.3 ± 8.0	< 0.01**	0.94	0.28	< 0.01**
	ROB	7.6 ± 6.2	4.2 ± 6.7	< 0.01**			
Ashworth	CTR	20.6 ± 9.8	1.4 ± 11.5	0.66	0.44	0.99	0.50
	ROB	17.1 ± 11.5	1.5 ± 13.7	0.61			
BAT timing	CTR	14.1 ± 3.7	− 2.2 ± 3.4	< 0.01**	0.05	< 0.01**	< 0.01**
	ROB	17.3 ± 3.6	− 4.8 ± 3.7	< 0.01**			
BAT quality	CTR	2.5 ± 1.1	0.6 ± 1.0	< 0.01**	0.35	0.20	< 0.01**
	ROB	2.1 ± 0.8	0.9 ± 0.8	< 0.01**			
BAT timing pinch tasks	CTR	15.0 ± 4.5	− 2.2 ± 4.3	< 0.01**	0.05	< 0.05*	< 0.01**
	ROB	18.8 ± 4.5	− 5.6 ± 4.4	< 0.01**			
BAT quality pinch task	CTR	2.6 ± 1.0	0.5 ± 1.0	< 0.01**	0.18	0.06	< 0.01**
	ROB	2.0 ± 0.7	0.9 ± 0.8	< 0.01**			
BAT timing power task	CTR	13.4 ± 3.3	− 2.5 ± 3.1	< 0.01**	0.12	< 0.05*	< 0.01**
	ROB	15.9 ± 3.7	− 4.1 ± 3.7	< 0.01**			
BAT quality power task	CTR	2.6 ± 1.0	0.4 ± 0.9	< 0.01**	0.25	0.08	< 0.01**
	ROB	2.2 ± 0.8	0.9 ± 0.8	< 0.01**			

Values for baseline and change are given as means ± standard deviations

At baseline, the two groups of patients did not statistically differ in terms of age, sex, months post-stroke and type of lesion. Statistical ANOVA tests were conducted on 22 subjects equally distributed between the Robotic and Control groups. The baseline and the change in the outcome measures (FMA, Modified Ashworth and BAT scales) detailed for the two experimental groups are reported in Table 3, together with the p-values of the complete ANOVA test and those of the planned contrasts.

Both groups reported significant improvements. More in detail, all enrolled patients, not depending on treatment, significantly improved in terms of FMA (*F*_*(1,20)*_ = *47.1, p* < *0.001*) and functional BAT assessment (BAT timing: *F*_*(1,20)*_ = *63.8, p* < *0.001; BAT quality F*_*(1,20)*_ = *29.6, p* < *0.01*), whereas the level of spasticity did not significantly change after therapy (Modified Ashworth scale, F_(1,20)_ = 0.454, p = 0.054).

**Fig. 4:**
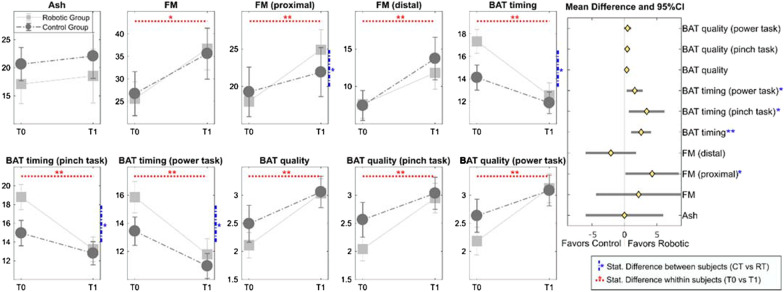
Change in clinical outcome measures for the two groups’ pre (T0) and post (T1) therapy (means and standard errors are reported). The forest plot in the right panel of the figure reports the differences of the changes in the two groups (the diamonds represent the averaged value and the horizontal lines are the 95%CI). The semi-plane on the right of the zero line indicates greater improvement for the Robotic Group whereas the left semi-plane indicates greater improvement for the Control group. Blue lines indicate statistically significant differences among the two groups, while red lines between enrollment and discharge

As regards the between group comparison, there were no significant differences at baseline between groups for any of the outcome measures. The graphical representation of the change in clinical outcome measures for the two groups is reported in Fig. 4. In particular, each panel reports the corresponding clinical test score pre (T0) and post (T1) therapy for the two groups (RG and CG) separately. Horizontal red dashed lines indicate a significant change between T0 and T1 not depending on the group. Vertical blue dashed line reports significant differences between the RG and CG groups. The right panel in Fig. 4 shows the forest plot of the clinical outcomes showing the confidence intervals of the differences between the two groups in terms of z-scores of the differences. Points aligned towards the right of the zero line indicate greater improvements in the Robotic Group and those aligned towards the left of the zero line indicate grater improvements in the Control Group.

Significant differences between groups in the change of the proximal portion of the FMA score and the BAT timing functional score were observed in favors of the RG. In particular, whereas the two groups were characterized by a similar averaged score at baseline for the proximal portion of the FMA (19.3 vs. 18.0 points for the control and the robotic group respectively), the robotic group recorded a significantly higher improvement with respect with the control group (6.9 ± 7.8 vs. 2.6 ± 10.9 points, see Table 3). The improvements in terms of functional ability measured as reduction of execution time in BAT scale, were significantly higher in the RG group than the CG group (p < 0.01), both for power and precision tasks (p < 0.05).

### Robotic outcomes

Since two patients were unable to autonomously complete the evaluation exercise described in Sect. 2.4.3 (i.e., without the robotic assistance), kinematics data from only 9 subjects out of 11 belonging to the Robotic group were analyzed.

**Fig. 5:**
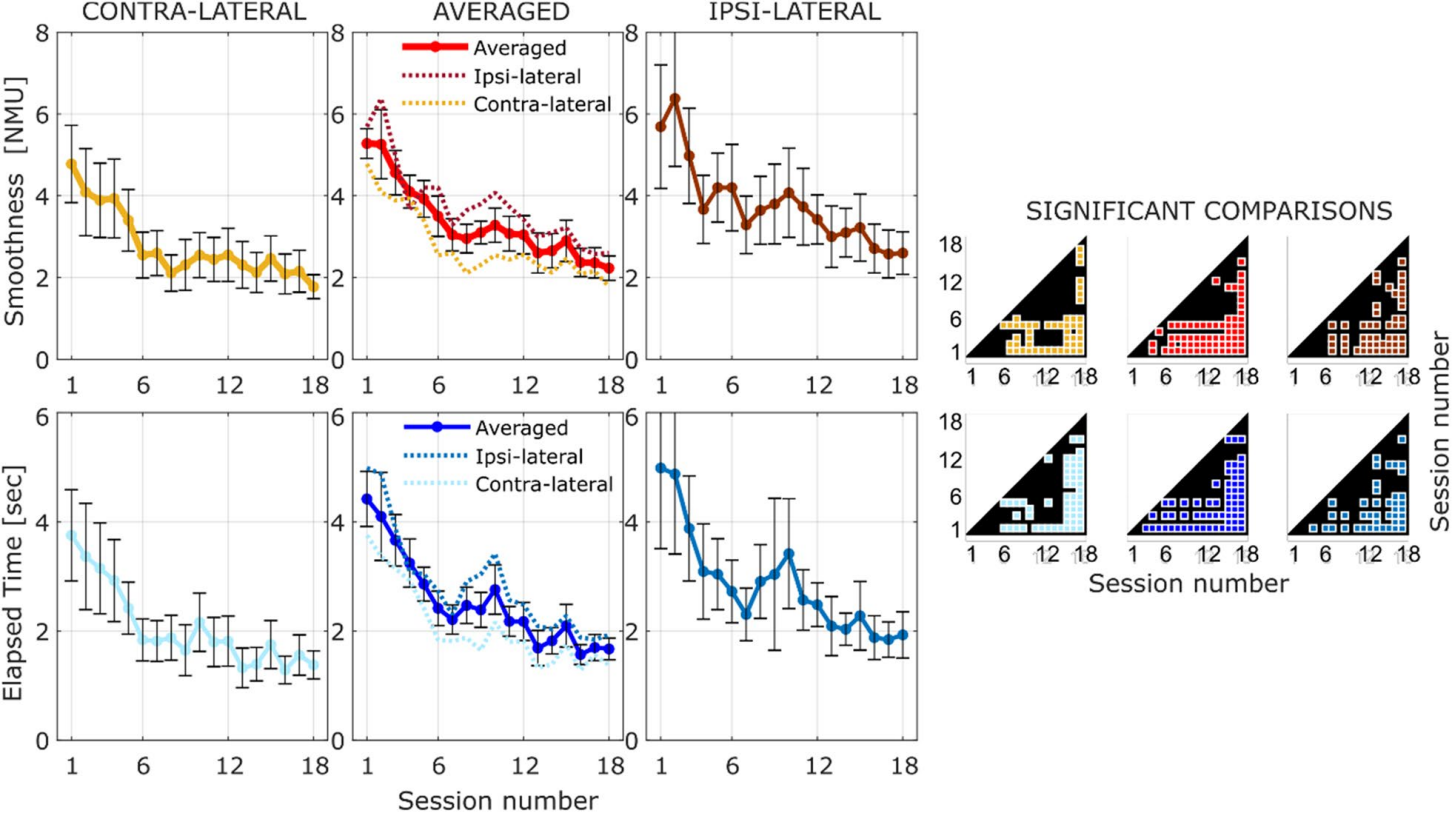
LEFT: Robotic performance indexes over sessions of the Robotic Group for all movements (central column), the ipsilateral movements (right column) and the contralateral movements (left column). The first row represents the smoothness in terms of number of movement units; the second row represents the time elapsed for accomplishing the movements. RIGHT: significance of the pairwise comparison across the 18 session. The colored spot marks the significant output of the t-test

Figure 5 shows the performance in terms of smoothness (first row) and completion time (second row) along the 18 rehabilitation sessions averaged over 9 subjects of the Robotic group. The three columns in the left panel represent the robotic measures divided for the contralateral and ipsilateral movements (first and last columns respectively) and the averaged performance (second column). For each robotic measure it is possible to note an improvement over time—decreasing of both the Number of Movement Units and the elapsed time for accomplishing the task. Significance between sessions is highlighted by the corresponding plots in the right panel of Fig. 5. In particular, colored markers highlight the rejection of the null hypothesis (p < 0.05) that the mean of a session is equal to the mean of another session (paired t-test with least significant difference procedure [29]).

Analogously to the clinical outcomes, statistical analysis was conducted using data belonging to the first session (pre-therapy) and the last session (post-therapy). The paired t-test shown both a marked decrease of movement time (*t*_*(8)*_ = *5.15*, *p* < *0.01*) and marked increase of smoothness quality expressed as number of peaks in the velocity profile (*t*_*(8)*_ = *5.01*, *p* = *0.01*).

**Fig. 6:**
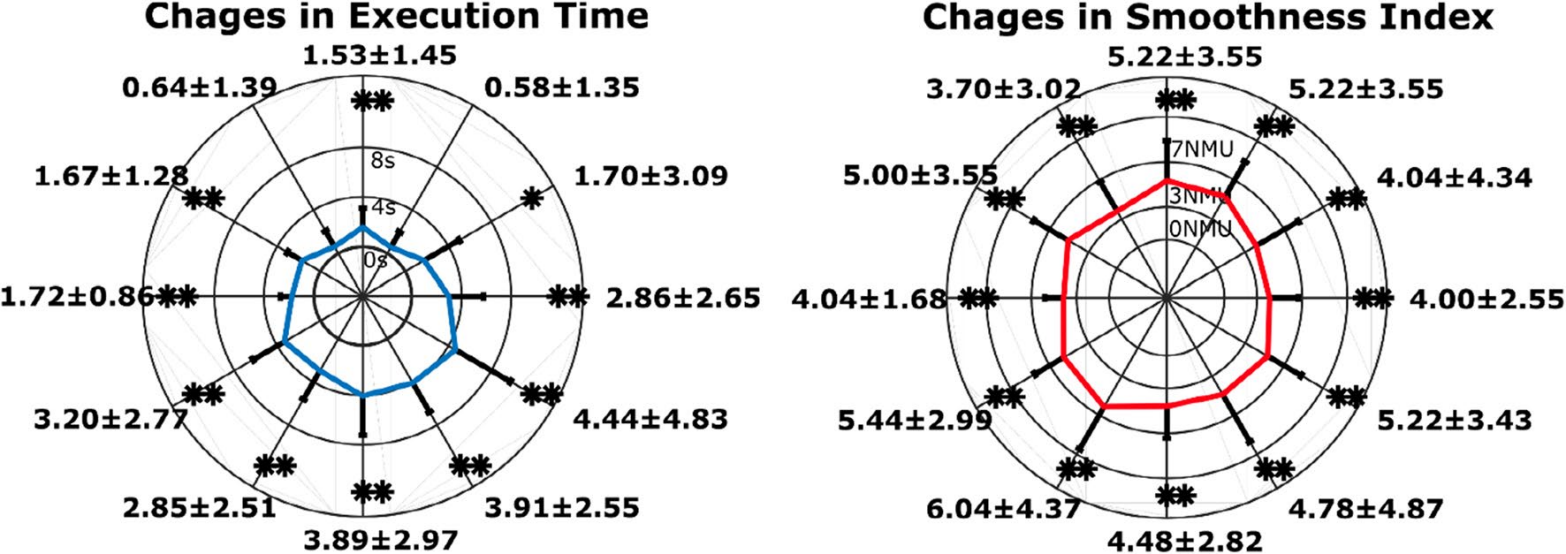
Automatic assessment of time execution (left) and smoothness of movement (right) over different directions in the vertical plane. The first and second radial plots show the difference for each direction of the first and the last rehabilitation sessions in terms of Execution Time and Smoothness Index respectively averaged over patients. For each direction ‘*’ and ‘**’ show significant differences (paired t-test) at p < 0.05 and p < 0.01 respectively

The performance improvement in movement execution was analyzed also over different directions of the vertical plane. In Fig. 6, the changes in performance between the first and the last rehabilitation session are reported for each direction. In particular, the amplitude for each direction is proportional to the change observed in that specific direction (i.e., a low amplitude represents a minimal change) and the asterisk mark a significant change between the first and last session (*p < 0.05 and **p < 0.01) assessed with the paired t-test.

### Correlation analysis

Multilinear regression analysis was conducted using as predictors variables the robotic measures measured at the baseline (completion time and smoothness divided by ipsi- and contra-lateral direction) and as response variable the change in FMA score pre and post the robotic treatment.

**Fig. 7:**
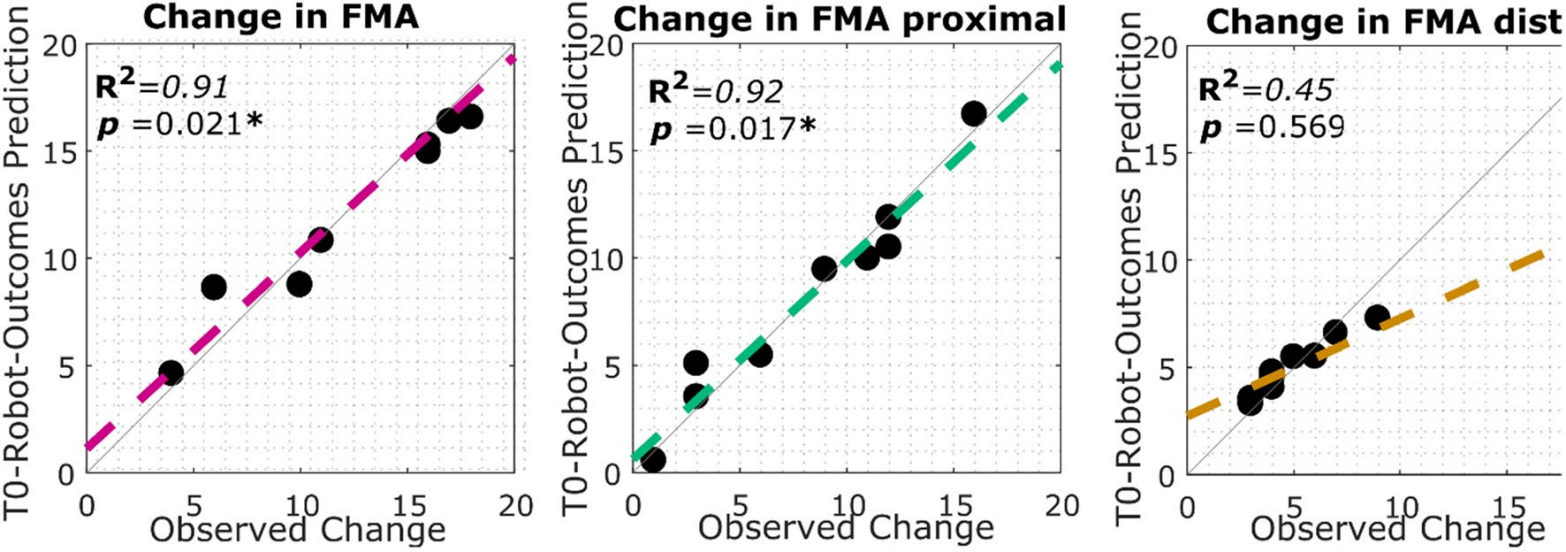
Multilinear regression analysis. Each point in the plot represent a patient of the RG. The x axis represents the observed change in the FM (left) in the proximal portion of the FM (central) and in the distal portion of the FM (distal). The y axis is the estimation of the change by using the robotic outcomes collected at the baseline (T0). The value coefficient of determination together with the p-value of the F statistic is reported for each graph

Obtained results for the FMA score, first panel of Fig. 7, evidenced a significant linear correlation between the robotic performance obtained at the baseline and the change in FMA score (*R*^*2*^ = *0.91*, *p* = *0.021*) using the following coefficients: ${t}_{ipsi}=-1.3; {t}_{contra}=-1.1; {s}_{ipsi}=-1.2;{ s}_{contra}=-1.6$.

Further analysis was conducted focusing on the proximal and distal portion of the FMA assessment scale. The model was able to significantly predict the change only in the proximal portion of the FMA (*R*^*2*^ = *0.92, p* = *0.017),* but not in the distal portion of the FMA (*R*^*2*^ = *0.45, p* = *0.569*).

## Discussion

According to previous studies conducted with robotic exoskeleton [11] we hypothesized that robotic treatment might lead to significant improvement in terms motor function domain if compared to conventional manual therapy. Considering the whole upper portion of the FMA, no significant difference was observed between the change in the CG and in the RG (8.9 ± 17.6 and 11.1 ± 13.9 respectively).

However, significant differences between the two treatment groups were found restricting the analysis to the proximal portion of the FMA scale. In particular, the Robotic Group improvement in the FMA-proximal portion of 6.9 ± 7.8 points was significantly higher than the 2.6 ± 10.9 points improvement of the Control Group. In fact, whereas the physical therapy includes a set of exercises focused on the rehabilitation of hand movement as well (e.g. place a pen to the side of the table and then grip it with the affected fingers), the robotic treatment conducted with the L-EXOS focused mainly on the rehabilitation of the arm functionality without requiring any particular task to be accomplished with hand/fingers movements. Considering the overall improvement in the FMA assessment, only 37% of it was represented by the distal portion within the RG, while 67% was represented by the proximal portion of the FMA. On the other hand, for the CG, the improvement in the distal portion achieved the 70% of the overall change in terms of FM against the 30% represented by the proximal portion.

On the other side, we expected to find improvements in functional outcome due to the execution of three-dimensional spatial training performed with robot exoskeleton assistance.

Interestingly, the two groups significantly differenced in the time execution of the BAT functional scale for both gross and fine manipulation tasks. In particular, the execution time in performing ADL tasks, measured through the functional BAT scale were significantly improved in the RG than the CG for both gross and fine movements tasks. This finding is in line with the study of Schaefer and colleagues [30], in which it was demonstrated that task-specific training activity could potentially generalize to a broader spectrum of motor tasks than the one practiced. In our case, the robot-mediated repetitive practice of the proposed functional task (pouring water out of a bottle into a set of glasses and cups), due to the purposefulness and the multi-step nature of the task itself, could have transferred to other untrained tasks such of those listed in BAT scale (e.g., moving a shoe box over another shoe box).

The observed difference between the two groups only in time execution of BAT scale, but not quality, can be explained by two reasons: (1) the measurements of a quantitative parameter such as time are more precise in detecting changes in task execution rather than qualitative parameters (2) the improvement of performance in ADLs might not be necessarily be due only to an overall improvement of quality of movement, since also the adoption of compensatory movement strategies play a role in function recovery.

The clinical and functional improvements mentioned above, are reflected in the robotic performance measured during the evaluation exercise in the RG sessions, which allowed to extract quantitative indexes of the quality of movements (execution time and smoothness).

In line with this finding, the regression analysis reported a significant predictive ability of the robotic outcomes measured at baseline (pre-treatment) and the change in the proximal portion of the FMA measured post-treatment. We can claim that the extracted robotic indices might serve as significant biomarkers for predicting clinical changes at the proximal segment of the upper limb after the proposed robot-mediated therapy.

A quantitative measure of the motion smoothness plays an important role in the evaluation of sensorimotor deficits and motor learning and can be used as a valid index of motor recovery in stroke patients [31]. The multiple pairwise comparisons between sessions (right panel in Fig. 5), viewed as hypothesis generating procedure [29], show an interesting trend that splits the proposed robotic therapy protocol in three phases of the motor learning process. The motor improvement rapidly increases after the first 6 sessions (phase 1) and then it becomes slower, reaching a plateau, with the increasing number of the sessions (phase 2, from session 6 to session 14). The third and last phase, approximately between session 14 and session 18, instead highlights a further improvement with respect to the other two previous phases.

An interesting finding is also the empirical ex-post observation of the time and number of sessions required for functional changes in upper limb task execution: from performance over time we can see how after 18 sessions of treatment a significant difference of performance is always reached in kinematic performance and a plateau is reached.

Within the proposed assessment exercise, we can evaluate also the change of performance along different directions. Analyzing isokinetic movements at various angular velocities within the capable range of motion for joints provides a valid tool to monitor the level of spasticity [32]: it has been found also that the coordination pattern of shoulder and elbow joints is preserved differently for reaching movements executed in the contra-lateral and ipsilateral space [11]. In this study, an approximately isotropic improvement of the smoothness along the twelve directions was observed, whereas, as regards the execution time, the improvement was higher for the movements towards the lower part of the plane than for the upper part of the plane. The isotropic change of smoothness in all directions of space is a good indicator that the rehabilitation training did not affect preferentially any direction of movement, while the reduced improvement in execution time in unsupported reaching movements performed “against gravity” can be related to the higher effort required for antigravity movements and to the onset of abnormal flexor synergies when increasing amounts of shoulder abduction torque are required to actively maintain the arm elevation [33]*.*

Although hand paths of stroke survivors during visually guided reaching planar tasks along different directions exhibit movement abnormalities [34], the progressive expression of loss of independent joint control cannot be observed in planar tasks only. During the execution of 3D movements also the alteration of proximal muscle synergy structure dominated by activation of shoulder muscles can be observed in stroke survivors with mild and moderate impairment [35].

For this reason it is relevant to see that observed correlation with the proximal portion of the FMA confirms the potential capability of exoskeletons to detect eventual changes in proximal muscle pattern and recruitment, that appears to play a major role in motor recovery in stroke.

Rosenthal et al. [36] recently proposed a method that exploit the usage of a robotic device for the performance-based identification of practiced movements that can reduce upper-limb motor impairment due to stroke. These kind of approaches, aimed at identifying robotic biomarkers strictly related to the motor recovery process, could allow the definition of individualized robotic rehabilitation protocols which may be effective in enhancing the functional outcome of a therapy [17, 37].

## Conclusions

The results of this study showed that both manual and robotic treatment can lead to significant improve in terms of FMA and BAT in chronic stroke patients. In particular, a significant greater improvement of the robotic treatment was observed in the proximal portion of the FMA and in the execution time of the BAT tasks. The robotic treatment showed also the double fold advantage of automatically extracting performance indexes for both monitoring the motor recovery process of each patient and to potentially predict the change in clinical score after the treatment.

## Data Availability

The datasets used and/or analysed during the current study are available from the corresponding author on reasonable request.

## References

[1] Cauraugh JH, Lodha N, Naik SK, Summers JJ (2010). Bilateral movement training and stroke motor recovery progress: a structured review and meta-analysis. Hum Mov Sci.

[2] Takeuchi N, Izumi S. Rehabilitation with poststroke motor recovery: a review with a focus on neural plasticity. Stroke Res Treat [Internet]. 2013;2013. 10.1155/2013/128641 PMC3659508 23738231

[3] Burdet E, Franklin DW, Milner TE. Human robotics: neuromechanics and motor control. MIT Press; 2013.

[4] Cameirão MS, Badia SBI, Duarte E, Frisoli A, Verschure PFMJ (2012). The combined impact of virtual reality neurorehabilitation and its interfaces on upper extremity functional recovery in patients with chronic stroke. Stroke.

[5] Bertani R, Melegari C, De Cola MC, Bramanti A, Bramanti P, Calabrò RS (2017). Effects of robot-assisted upper limb rehabilitation in stroke patients: a systematic review with meta-analysis. Neurol Sci.

[6] Mehrholz J, Pohl M, Platz T, Kugler J, Elsner B. Electromechanical and robot-assisted arm training for improving activities of daily living, arm function, and arm muscle strength after stroke. Cochrane Libr. 2015; 10.1002/14651858.CD006876.pub4 PMC6465047 26559225

[7] Rodgers H, Bosomworth H, Krebs HI, van Wijck F, Howel D, Wilson N (2019). Robot assisted training for the upper limb after stroke (RATULS): a multicentre randomised controlled trial. Lancet.

[8] Frisoli A, Solazzi M, Loconsole C, Barsotti M (2016). New generation emerging technologies for neurorehabilitation and motor assistance. Acta Myol.

[9] Reinkensmeyer DJ, Wolbrecht ET, Chan V, Chou C, Cramer SC, Bobrow JE (2012). Comparison of three-dimensional, assist-as-needed robotic arm/hand movement training provided with Pneu-WREX to conventional tabletop therapy after chronic stroke. Am J Phys Med Rehabil.

[10] Klamroth-Marganska V, Blanco J, Campen K, Curt A, Dietz V, Ettlin T (2014). Three-dimensional, task-specific robot therapy of the arm after stroke: a multicentre, parallel-group randomised trial. Lancet Neurol.

[11] Frisoli A, Procopio C, Chisari C, Creatini I, Bonfiglio L, Bergamasco M (2012). Positive effects of robotic exoskeleton training of upper limb reaching movements after stroke. J Neuroeng Rehabil.

[12] Lee S, Park G, Cho D, Kim H, Lee J, reports SK-S, et al. Comparisons between end-effector and exoskeleton rehabilitation robots regarding upper extremity function among chronic stroke patients with moderate-to. nature.com [Internet]. 10.1038/s41598-020-58630-2 PMC7000418 32019981

[13] Milot M-H, Spencer SJ, Chan V, Allington JP, Klein J, Chou C (2013). A crossover pilot study evaluating the functional outcomes of two different types of robotic movement training in chronic stroke survivors using the arm exoskeleton BONES. J Neuroeng Rehabil.

[14] Jeffers MS, Karthikeyan S, Gomez-Smith M, Gasinzigwa S, Achenbach J, Feiten A (2018). Does stroke rehabilitation really Matter? Part B: an algorithm for prescribing an effective intensity of rehabilitation. Neurorehabil Neural Repair.

[15] Stinear CM (2017). Prediction of motor recovery after stroke: advances in biomarkers. Lancet Neurol.

[16] Boyd LA, Hayward KS, Ward NS, Stinear CM, Rosso C, Fisher RJ (2017). Biomarkers of stroke recovery: consensus-based core recommendations from the Stroke Recovery and Rehabilitation Roundtable. Int J Stroke.

[17] Krebs HI, Peltz AR, Berkowe J, Angacian G, Cortes M, Edwards D. Robotic biomarkers in RETT Syndrome: Evaluating stiffness. In: 2016 6th IEEE International Conference on Biomedical Robotics and Biomechatronics (BioRob). IEEE; 2016. p. 680–4.

[18] Patel S, Park H, Bonato P, Chan L, Rodgers M (2012). A review of wearable sensors and systems with application in rehabilitation. J Neuroeng Rehabil.

[19] Krebs HI, Krams M, Agrafiotis DK, DiBernardo A, Chavez JC, Littman GS (2014). Robotic measurement of arm movements after stroke establishes biomarkers of motor recovery. Stroke.

[20] Mostafavi S, Mousavi P, Dukelow S, Scott S. Robot-based assessment of motor and proprioceptive function identifies biomarkers for prediction of functional independence measures. J Neuroeng Rehabil [Internet]. 2015; 12(1). 10.1186/s12984-015-0104-7 PMC4661950 26611144

[21] Douiri A, Grace J, Sarker S, Tilling K, McKevitt C, Wolfe C (2017). Patient-specific prediction of functional recovery after stroke. Int J Stroke [Internet]..

[22] Harvey R (2015). Predictors of functional outcome following stroke. Phys Med Rehabil Clin N Am [Internet]..

[23] Kim J, Shin W (2014). How to do random allocation (randomization). Clin Orthop Surg.

[24] Barreca S, Wolf SL, Fasoli S, Bohannon R (2003). Treatment interventions for the paretic upper limb of stroke survivors: a critical review. Neurorehabil Neural Repair.

[25] Sallés L, Martín-Casas P, Gironès X, Durà MJ, Lafuente JV, Perfetti C (2017). A neurocognitive approach for recovering upper extremity movement following subacute stroke: a randomized controlled pilot study. J Phys Ther Sci [Internet]..

[26] Frisoli A, Rocchi F, Marcheschi S, Dettori A, Salsedo F, Bergamasco M. A new force-feedback arm exoskeleton for haptic interaction in virtual environments. In: Eurohaptics Conference, 2005 and Symposium on Haptic Interfaces for Virtual Environment and Teleoperator Systems, 2005 World Haptics 2005 First Joint. 2005. p. 195–201.

[27] Frisoli A, Sotgiu E, Procopio C, Bergamasco M, Rossi B, Chisari C. Design and implementation of a training strategy in chronic stroke with an arm robotic exoskeleton. In: 2011 IEEE International Conference on Rehabilitation Robotics. IEEE; 2011. p. 1–8. 10.1109/ICORR.2011.5975512 22275708

[28] Rohrer B, Fasoli S, Krebs HI, Hughes R, Volpe B, Frontera WR (2002). Movement smoothness changes during stroke recovery. J Neurosci.

[29] Saville DJ (1990). Multiple comparison procedures: the practical solution. Am Stat.

[30] Schaefer SY, Patterson CB, Lang CE (2013). Transfer of training between distinct motor tasks after stroke: implications for task- specific approaches to upper extremity neurorehabilitation. Neurorehabil Neural Repair.

[31] van Kordelaar J, van Wegen E, Kwakkel G (2014). Impact of time on quality of motor control of the paretic upper limb after stroke. Arch Phys Med Rehabil [Internet]..

[32] Nam HS, Koh S, Kim YJ, Beom J, Lee WH, Lee S-U (2017). Biomechanical reactions of exoskeleton neurorehabilitation robots in spastic elbows and wrists. IEEE Trans Neural Syst Rehabil Eng.

[33] Ellis MD, Lan Y, Yao J, Dewald JPA (2016). Robotic quantification of upper extremity loss of independent joint control or flexion synergy in individuals with hemiparetic stroke: a review of paradigms addressing the effects of shoulder abduction loading. J Neuroeng Rehabil.

[34] Dukelow SP, Herter TM, Bagg SD, Scott SH (2012). The independence of deficits in position sense and visually guided reaching following stroke. J Neuroeng Rehabil.

[35] Roh J, Rymer W, Beer R. Evidence for altered upper extremity muscle synergies in chronic stroke survivors with mild and moderate impairment. Front Hum Neurosci [Internet]. 2015;9(FEB). 10.3389/fnhum.2015.00006 PMC4324145 25717296

[36] Rosenthal O, Wing AM, Wyatt JL, Punt D, Brownless B, Ko-Ko C (2019). Boosting robot-assisted rehabilitation of stroke hemiparesis by individualized selection of upper limb movements—a pilot study. J Neuroeng Rehabil.

[37] Semrau JA, Herter TM, Scott SH, Dukelow SP (2015). Examining differences in patterns of sensory and motor recovery after stroke with robotics. Stroke.

